# Virtual Town Hall Meetings to Convey Emergency Medicine Residency Program Information to Students

**DOI:** 10.5811/westjem.2022.4.54637

**Published:** 2022-07-13

**Authors:** Geoffrey Comp, Kateland Townley, Eric Blazar, Taylor Webb, Mark Keuchel, Bikash Bhattarai, Amrita Vempati, Michael Epter, Katherine Holmes

**Affiliations:** * Creighton University School of Medicine, Valleywise Health Medical Center, Department of Emergency Medicine, Phoenix, Arizona; † Inspira Health Network, Department of Emergency Medicine, Vineland, New Jersey; ‡ INTEGRIS Southwest Medical Center, Department of Emergency Medicine, Oklahoma City, Oklahoma; ¶ John Peter Smith Hospital, Department of Emergency Medicine, Fort Worth, Texas; § Valleywise Health Medical Center, Department of Research, Phoenix, Arizona

## Abstract

**Background:**

Applying to emergency medicine (EM) residency programs as a medical student is challenging and complicated in a normal year, but the 2020/2021 application cycle was further complicated by the COVID-19 pandemic. Due to the decrease of in-person opportunities for students to connect with residency programs, virtual “town-hall” meetings were developed. In this study our primary objective was to determine whether attendance at a virtual residency program information session improved the perceived knowledge of curriculum information and program exposure to medical students applying to an EM residency.

**Methods:**

Four study sites hosted a total of 12 virtual events consisting of residents, faculty, or both. Standardized pre-event/post-event surveys were conducted to capture medical student perceptions before/after each of the virtual sessions. Apart from measuring the improvement in students’ perceived knowledge of a program by gauging their responses to each question, we used a 10-question composite score to compare pre- vs post-event improvement among the participants.

**Results:**

The pre-event survey was completed by 195 attendees, and the post-event survey was completed by 123 attendees. The median and mean composite score to this 10-question survey improved from 32.19 to 45, and 31.45 to 44.2, respectively, in the pre- to post-event survey.

**Conclusion:**

This study showed improvement of medical students’ perceived knowledge of residency programs (reflected as increased agreement from pre- to post-event survey). The data demonstrates through question responses that students not only obtained information about the programs but also were able to gain exposure to the culture and “feel” of a program. In a non-traditional application season in which students are unable to pursue their interest in a program through audition rotations, virtual town hall events, along with other asynchronous events, may be a reasonable approach to increasing medical student understanding and awareness of a program and its culture.

## INTRODUCTION

Applying to residency is a challenging annual endeavor for medical students who are forced to make decisions about where they would like to train when they may have a limited amount of information about programs. They rely on residency websites, social media, personal communication, online forums, and in emergency medicine (EM) in particular, audition rotations and in-person visits.^1^ These opportunities serve both as a pre-interview experience for the prospective student and as a place for information exchange. Students discuss and experience culture, residents, attending physicians, patient populations, support staff, and the physical location of the programs. Programs attempt to identify whether the student is a fit with the culture and gauge their ability to learn and grow, as well as their baseline medical knowledge. It has been shown that some of the most important factors in student selection of a program include how happy the residents seem, faculty-resident relationships, how well the residents work together, values of residents and faculty, and whether the residents spend time together outside of the residency and there are shared interests.^2^ These are traits difficult to communicate without in-person experiences.

During the 2020–2021 application cycle, amidst the coronavirus 2019 (COVID-19) global pandemic, most of the opportunities that previously took place in person were not feasible. There was significant concern that many third-year medical students would not be able to gain clinical and program-specific experience before submitting their residency applications.^3^ Fewer and shorter clinical rotations were available for interested students because host institutions were attempting to limit COVID-19 exposure. COVID-19 also led to the creation of the “Consensus Statement on the 2020–2021 Residency Application Process for US Medical Students Planning Careers in Emergency Medicine in the Main Residency Match” by 10 medical education organizations, limiting each student to one EM rotation, typically at their home institution.^4^ Both residency programs and medical students worked to develop creative ideas to showcase their strengths and connect with each other through innovative methods not previously used. Students were attempting to gather information and evaluate programs while residency programs were working to identify alternative ways to share more than the facts and figures that websites typically offer.

One novel method emerged for students and residents to share information through virtual “meet and greet” events, sometimes referred to as “town hall events,” “get to know us sessions,” or “virtual tours.” These virtual events, hosted by the residency program, became a popular way to connect programs and students. The basic format was a series of sessions, or a single video session, hosted and promoted by a specific residency program, led by either current residents, faculty, or both. A brief presentation or introduction was typically provided, followed by a space for students to ask questions and glean information about specific program details. Some programs offered multiple sessions, each highlighting specific strengths.

*Population Health Research Capsule*

What do we already know about this issue?

*Residencies use virtual “meet and greet” events to connect programs and students, but the effectiveness of these events has not been studied.*

What was the research question?

*Do virtual information sessions improve medical student knowledge of curriculum offerings and provide enough program exposure?*

What was the major finding of the study?

*Attendance at virtual information sessions improves the perceived knowledge of curriculum and enhances program exposure.*

How does this improve population health?

*Residency programs should consider incorporating these types of events to enhance medical student recruitment and convey important program information.*

After performing a brief search through social media outlets, program websites, and virtual newsletters from EM professional organizations, we found that as of August 2020 at least 99 of 273 EM residency programs publicized or participated in a virtual event. This number significantly increased by the end of the 2020–2021 application cycle with 224 EM residency programs participating in a virtual event as of March 2021. These events ranged from Instagram or Facebook live events to Zoom gatherings and panels. Prior to this application cycle, such events were uncommon and did not appear to be a standard way of disseminating information to the residency applicant pool. Thus, there was no published research to date on the effectiveness of a live virtual recruitment event in providing useful information to EM applicants attempting to make residency selection decisions. With many EM residency programs transitioning to these virtual events for recruitment purposes, we believed it would be important to determine whether a virtual format is an effective method of improving applicant understanding of curriculum information and enhancing program exposure.

In this study our primary objective was to evaluate the usefulness of this kind of event at four different residencies within the US using pre- and post-event surveys. Specifically, does attendance at a virtual residency program information session improve the perceived knowledge of curriculum information and program exposure to medical students applying to an EM residency? We hypothesized that participation would allow for increased self-reported knowledge of various specific program details and increase the interest of the applicants in the program.

## METHODS

This was a multicenter pre- and post-intervention survey of medical students attending a virtual residency information session at one of the four study sites. Site one is a three-year county hospital-based EM program with 45 residents in Arizona, which hosted one session. Site two is a community-based four-year EM program in Oklahoma with 30 residents, which hosted two sessions. Site three is a three-year county hospital-based EM program with 45 residents in Texas that hosted eight sessions. Finally, site four is a three-year community rural ED with 36 residents in New Jersey that hosted one session.

Each site was responsible for promoting its own event including advertising, date selection, time, virtual software platform used, and number of dates to offer their meetings. A standardized pre- and post-event survey was used by all four sites with voluntary participation by the students who attended each event. We provided each program with a survey template using Google Forms (Google LLC. Mountain View, CA), which each program could then individually brand without changing the content of the survey. Students were recruited before and after the session to fill out the survey information.

As the use of virtual information sessions is a relatively novel practice, there are no previously validated surveys on this topic. Students were instructed to indicate their agreement using a Likert scale with specific statements before and after the sessions. Questions were developed, reviewed, piloted, and refined by the research team and adapted from previously published literature indicating some of the top reasons medical students select a residency program.^2^,^5^ Written consent was obtained prior to completion of the surveys by all subjects. We also collected demographic information was also collected including age, gender, medical school year of training, and geographic location. To ensure blinding and total anonymity, the last five digits of the participants’ phone numbers were used to link the pre- and post-event surveys. It was imperative to make it clear to the participants that the survey was both voluntary and anonymous to prevent concern about possible attribution or effect on their residency application. The project received a human subjects’ research exemption from the Valleywise Health Institutional Review Board and was completed in 2020.

Summary of all the respondents and questions were described along with comparison of pre-post matched responses linked using the last five digits of the respondents’ phone numbers. For each question, the highest level of agreement was assigned a score of 5 with the lowest level of agreement assigned as 1. We used the Wilcoxon signed-rank test to compare the score for each question by students who responded to both pre- and post-surveys. Responses were further dichotomized into “agree or strongly agree” vs all other responses; we used McNemar two-sided tests to evaluate changes in agreement pre- and post-surveys. For 10 questions, perfect favorable agreement would make a total agreement score of 50, which was used as a composite score to evaluate overall pre-post change in level of agreement. We used the Wilcoxon signed-rank test to compare differences in composite scores pre- and post-survey.

## RESULTS

Overall, the pre-survey was completed by 195 attendees, and the post-event survey was completed by 123 attendees. Response proportions for pre-surveys ranged from 40.23% to 59.02% of total attendance across four locations, which were slightly higher than 21.88–49.18% for the post-event surveys (Table 1). Survey response and demographic information is displayed in Table 2, including a breakdown of which location session the participants attended. Distribution of those participants who chose to report gender was nearly evenly split between male and female. Many of the participants were between the ages of 26–30, and most were fourth-year medical students. Geographically, all areas of the country were represented with the largest number from the southwest United States and the lowest percentage of participants from the northwest or international.

Table 3 illustrates pre- and post-event survey summaries. Values ranged from 1 to 5 with 1 representing strongly disagree, and 5 representing strongly agree. The median and mean composite score to this 10-question survey improved from 32.19 to 45, and 31.45 and 44.2, respectively, in the pre- to post-event surveys. Of the total number of completed surveys 75 attendees from three institutions were matched as completing both the pre- and post-event surveys (35 from site one, 24 from site two, and 16 from site three). Unfortunately, at site four, no participants could be matched to link the pre- and post-event surveys.

We further analyzed these 75 participant responses to determine efficacy of the goals of the information sessions. Table 4 shows overall data results for these 75 matched responses, as well as location-specific results. All questions showed improvement from pre- to post-event surveys following the information sessions. Table 5 displays the matched pre- and post-event question results and demonstrates a change from before and after the virtual information session. For statistical analysis, “neutral,” “disagree,” and “strongly disagree” (1, 2 and 3) were clustered as a disagreement. Similarly, “agree,” and “strongly agree,” were clustered as an agreement to improve overall data analysis. Of note, 15 responses were missing from question one due to site four inadvertently editing their version of the survey, removing the question. These were removed for data analysis. McNemar *P*-values were < 0.001 for all 10 questions.

## DISCUSSION

The ways in which residency programs and potential applicants interact will continue to change as the COVID-19 pandemic fluctuates and resolves. In this study we were able to show improvement toward agreement from pre- to post-event surveys after virtual recruitment events. The data clearly demonstrates through the students’ responses to questions that they not only obtained information about the program (number of elective rotations, general curriculum, emergency department layout, presence of specialty tracks, fellowship potential), but also information regarding the culture and “feel” of a program, which can sometimes be challenging to convey. Additionally, the results demonstrate that students were not only provided with tools for contacting program members, but that they were comfortable doing so after attending one of these sessions. As this study was designed to identify the perceived benefit of a virtual information session, further research opportunities include directly assessing applicant knowledge of residency-specific details.

As new techniques and ideas are developed to navigate a new virtual normal, it is important to ensure that students can make appropriate decisions about where they would like to match for residency. Many of these virtual sessions were developed to address the 2020–2021 travel and visiting restrictions. However, this type of event will likely be beneficial in the future as well. These virtual gatherings can continue to be used as a tool by programs to help showcase their culture, community, and strengths. They can also provide an opportunity for students who are unable to physically visit programs gain more information about a residency program.

## LIMITATIONS

We identified various limitations throughout this research experience. The most significant limitation was the low response rate for the surveys. As the design included a pre- and post-event survey with no requirements for completion, there was a large discrepancy between the number of students who registered, completed the pre-event survey, and attended the event, and the number of students who completed the post-event survey. There were fewer participants for the post-surveys relative to the pre-surveys across each of the four locations resulting in fewer matchable pairs available for the pre-post response comparisons. Unfortunately, data was missing or incomplete due to the survey nature, and not all pre- and post-event survey responses could be matched, resulting in some incomplete data. As a result, we are only able to report results from students willing to complete both surveys. Due to the small data sample size, many of the numerical results may be impacted, and there is a potential for variation in the results. However, our limited data on the responses to questions indicated a dramatic improvement in perceived knowledge, with many areas showing considerable advancement in perceived knowledge of the program. While the individual percentage of improvement could change with a larger sample size, it is unlikely that the overall benefit of virtual information sessions for interested medical students would be rejected.

During the design of the protocol, we determined that comfort of the participants was paramount, as we did not want there to be undue pressure for a student to complete the survey or to feel that there would be a professional consequence associated with participation. Students received one email before the session and one after recruiting them to participate in the survey with no other correspondence, which likely hindered our response rate. Future studies and follow-up questionnaires regarding this topic may improve response rates by sending additional follow-up correspondence. One option would be to use a staff member not associated with the residency to send additional emails and collect responses to maintain objectivity, resulting in another layer of protection for students completing the questionnaire.

Additionally, there was no standardized structure on what was to be included at each of the study site information sessions. Each location was free to promote, advertise, and host its own session or multiple sessions without specific oversight or specific requirements. This was intentionally done to allow for the creativity, personality, and innovation of each program to shine through without external modification. While we emphasized both in writing and verbally our goal of anonymity, some students may have felt pressured to respond to the surveys in a positive nature as they were likely applying for a residency position with the program. Furthermore, as students were self-selecting which information sessions to attend, there is a potential for a positive selection bias. Finally, as these programs were recruiting in the same application cycle, there may have been attendance overlap by medical students participating in the individual surveys. Future studies on this topic may benefit from including sites from different geographic locations and with a wider range of program sizes to further strengthen results.

Additionally, our study questions were targeted for short-term follow-up and did not prove increased familiarity long-term or necessarily affect the program’s recruitment of candidates. Additional studies including how attendance at a virtual information session may affect a student’s rank list or match results would be areas of future investigation to further examine the benefit of virtual sessions.

## CONCLUSION

It is important for medical students and EM residency programs to be able to critically evaluate each other to allow for the best possible match. Hosting virtual meet-and-greet events can be an effective way for programs to attempt to facilitate this type of information exchange when in-person interaction is limited. Attendance at a virtual residency program information session appears to improve the perceived knowledge of curriculum information and program exposure to medical students applying to an EM residency. Residency programs should consider incorporating these types of events to enhance medical student recruitment and convey important program information.

## Figures and Tables

**Table 1:** Response proportions to surveys administered before and after virtual information sessions across the four study locations.

Location	Attendance	Count of respondents* (Response percentage)	

		Pre-survey	Post-survey
Site 1	151	69 (45.7)	41 (27.15)
Site 2	87	35 (40.23)	31 (35.63)
Site 3	61	36 (59.02)	30 (49.18)
Site 4	96	51 (53.13)	21 (21.88)
Overall	395	191 (48.35)	123 (31.14)

* Actual count of responses for each question may be less than the total due to non-response to that question.

**Table 2:** Respondent demographics.

	Count	Percentage
Gender		
Male	72	36.92
Female	65	33.33
***Missing***	58	29.74
Age range		
26–30	113	57.95
20–25	39	20.00
31–35	32	16.41
36–40	6	3.08
41–45	1	0.51
***Missing***	4	2.05
Year of training		
MS4	150	76.92
MS3	21	10.77
MS2	8	4.10
MS1	7	3.59
Graduate	4	2.05
***Missing***	5	2.56
Geographic location		
Southwest USA	55	28.21
Northeast USA	48	24.62
Southeast USA	38	19.49
Central USA	34	17.44
Northwest USA	11	5.64
International	5	2.56
***All other values***	4	2.05
Completed pre-surveys		
Site 1	69	35.38
Site 2	35	17.95
Site 3	36	18.46
Site 4	51	26.15
***Missing***	4	2.05
Completed post-surveys		
Site 1	41	33.33
Site 2	21	17.07
Site 3	30	24.39
Site 4	31	25.20

*MS*, medical student; *USA*, United States of America.

**Table 3:** Knowledge score comparison before and after the virtual town hall meetings with medical students (n = 75 pre-post paired responses).

	Pre-score				Post-score				
	
Questions	Min	Mean	Median	Max	Min	Mean	Median	Max	P*
I am familiar with the general curriculum of the program.	1	3.19	3	5	2	4.35	4	5	<0.001
I am familiar with the number of elective rotations at the program.	1	2.84	3	5	2	4.2	4	5	<0.001
I am familiar with the general layout of the ED.	1	2.44	2	5	2	3.94	4	5	<0.001
I am aware of the different specialty tracks offered at the program.	1	2.68	2	5	2	4.14	4	5	<0.001
I am aware of the different post-residency fellowships offered by the program.	1	2.67	2	5	2	4.17	4	5	<0.001
I know what types of recreational activities I can experience in AZ.	1	3.36	4	5	1	4.43	5	5	<0.001
I have a good understanding of the culture of the residency community.	1	2.85	3	5	3	4.59	5	5	<0.001
I am interested in applying for a residency position at this program.	1	4.48	5	5	4	4.87	5	5	0.0011
If I have questions about the program, I am comfortable reaching out to a member of the program.	1	3.93	4	5	3	4.76	5	5	<0.001
I have contact information for members of the program and residency leadership to allow for further discussion.	1	3	3	5	2	4.73	5	5	<0.001
Composite score (maximum possible is 50 using all 10 questions).	10	31.45	32.19	50	30	44.2	45	50	<0.001

Values corresponding to the level of agreement: 1 = Strongly disagree, 2 = Disagree, 3 = Neutral, 4 = Agree, 5 = Strongly agree

* Wilcoxon signed-rank P-value based on the students who responded to both pre- and post-surveys.

*ED*, emergency department; *AZ*, Arizona.

**Table 4:** Composite scores for 75 respondents who responded to both pre- and post-surveys

Composite score (Maximum possible = 50)		Pre-survey	Post-survey	P
Overall				
All respondents	N	75	75	
	Mean	32.70	45.04	<.0001
	Median	33.00	45.00	
	Min	11.00	36.00	
	Max	49.00	50.00	
Location				
Site 1	N	35	35	
	Mean	32.57	46.23	<.0001
	Median	33.00	47.00	
	Min	11.00	41.00	
	Max	47.00	50.00	
Site 2	N	24	24	
	Mean	32.92	43.75	<.0001
	Median	32.50	44.50	
	Min	24.00	36.00	
	Max	49.00	50.00	
Site 3	N	16	16	
	Mean	32.68	44.38	<.0001
	Median	33.30	44.50	
	Min	19.98	39.00	
	Max	42.18	49.00	

As one of the locations inadvertently removed the first question in their survey, the corresponding result was calculated based on agreement score for the rest of questions, which resulted into a non-integer number.

**Table 5:** Dichotomized levels of agreement of the respondents to the questions before and after the intervention.

		Post-intervention response, count (percentage)			P*
		
	Pre-intervention response, count (percentage)	Neutral, disagree, or strongly disagree	Agree or strongly agree	Total	
Question 1: I am familiar with the number of elective rotations at the program; n (%).	Neutral, disagree, or strongly disagree	1 (1.33)	23 (30.67)	24 (32)	<0.001
	Agree or strongly agree	0	36 (48)	36 (48)	
	Missing response (excluded for analysis)	1 (1.33)	14 (18.67)	15 (20)	
	Total	2 (2.67)	97.33	100	
Question 2: I am familiar with the number of elective rotations at the program; n (%).	Neutral, disagree, or strongly disagree	6 (8)	38 (50.67)	44 (58.67)	<0.001
	Agree or strongly agree	1 (1.33)	30 (40)	31 (41.33)	
	Total	7 (9.33)	68 (90.67)	75 (100)	
Question 3: I am familiar with the general layout of the ED; n (%).	Neutral, disagree, or strongly disagree	15 (20)	46 (61.33)	61 (81.33)	<0.001
	Agree or strongly agree	0 (0)	14 (18.67)	14 (18.67)	
	Total	15 (20)	60 (80)	75 (100)	
Question 4: I am aware of the different specialty tracks offered at the program; n (%).	Neutral, disagree, or strongly disagree	7 (9.33)	46 (61.33)	53 (70.67)	<0.001
	Agree or strongly agree	0 (0)	22 (29.33)	22 (29.33)	
	Total	7 (9.33)	68 (90.67)	75 (100)	
Question 5: I am aware of the different post-residency fellowships offered by the program; n (%).	Neutral, disagree, or strongly disagree	6 (8)	41 (54.67)	47 (62.6)	<0.001
	Agree or strongly agree	1 (1.33)	27 (36)	28 (37.33)	
	Total	7 (9.33)	68 (90.67)	75 (100)	
Question 6: I know what types of recreational activities I can experience in the city; n (%).	Neutral, disagree, or strongly disagree	1 (1.33)	24 (32)	25 (33.33)	<0.001
	Agree or strongly agree	0 (0)	50 (66.67)	50 (66.67)	
	Total	1 (1.33)	74 (98.67)	75 (100)	
Question 7: I have a good understanding of the culture of the residency community; n (%).	Neutral, disagree, or strongly disagree	3 (4)	50 (66.67)	53 (70.67)	<0.001
	Agree or strongly agree	1 (1.33)	21 (28)	22 (29.33)	
	Total	4 (5.33)	71 (94.67)	75 (100)	
Question 8: I am interested in applying for a residency position at this program; n (%).	Neutral, disagree, or strongly disagree	0 (0)	5 (6.67)	5 (6.67)	<0.001
	Agree or strongly agree	0 (0)	70 (93.33)	70 (93.33)	
	Total	0 (0)	75 (100)	75 (100)	
Question 9: If I have questions about the program, I am comfortable reaching out to a member of the program; n (%).	Neutral, disagree, or strongly disagree	1 (1.33)	21 (28)	22 (29.33)	<0.001
	Agree or strongly agree	0 (0)	53 (70.67)	53 (70.67)	
	Total	1 (1.33)	74 (98.67)	75 (100)	
Question 10: I have contact information for members of the program and residency leadership to allow for further discussion; n (%).	Neutral, disagree, or strongly disagree	0 (0)	36 (48)	36 (48)	<0.001
	Agree or strongly agree	0 (0)	39 (52)	39 (52)	
	Total	0 (0)	75 (100)	75 (100)	

* McNemar test.
